# First MCL-1-selective BH3 mimetics as potential therapeutics for targeted treatment of cancer

**DOI:** 10.1038/cddis.2015.168

**Published:** 2015-07-09

**Authors:** S Besbes, C Billard

**Affiliations:** 1 INSERM U965, Hôpital Lariboisière, Paris, France; 2 Université Paris Diderot-Paris 7, Paris, France

In a recent issue of *Cell Death and Disease*, Leverson *et al.*^1^ have identified for the first time several potent and selective small-molecules MCL-1 inhibitors displaying on-target cellular activity and inducing mitochondrial apoptosis in cancer cells. The discovery of these inhibitors exemplified by the compound A-1210477 constitutes a milestone in the development of MCL-1-specific BH3 mimetics for anticancer therapy.

Prosurvival members of the BCL-2 family such as BCL-2, BCL-X_L_ and MCL-1 are overexpressed in many cancers.^2^ The antiapoptotic activity of these proteins is to bind and sequester their propapoptotic counterparts, notably the effectors of apoptosis BAX and BAK. Targeting the prosurvival BCL-2 family proteins therefore appeared as an attractive strategy to improve cancer therapy. One of the approaches to identify inhibitors of the prosurvival proteins has been to design small molecules capable of mimicking the BH3 domain of BH3-only BCL-2 family members.^3, 4^ The latter are indeed antagonists of the prosurvival members. The so-called BH3 mimetics include short BH3 peptides and organic molecules. Their prototype ABT-737 and its orally bioavailable derivative navitoclax bind with high affinity (in the nM range) to BCL-2, BCL-X_L_ and BCL-W, inhibit their activity and induce BAX and BAK-dependent apoptosis in tumor cells.^5, 6^ Navitoclax displays significant therapeutic effects especially in patients with hematologic malignancies but with a dose-limiting thrombocytopenia resulting from inhibition of BCL-X_L_ (that is a prosurvival factor for platelets).^7^ The BCL-2-specific derivative ABT-199 (venetoclax) was further designed.^8^ This agent has already shown impressive clinical results while sparing platelets, and is currently in phase III trials.^7^ Other authentic BH3 mimetics have been recently identified such as BM-1197, that displays characteristics similar to those of navitoclax, and the BCL-X_L_-selective A-1155463.^9, 10^

None of these compounds can bind and antagonize MCL-1, whereas this unique prosurvival BCL-2 family protein is known to have a crucial role in the development of many cancers and/or their resistance to chemotherapy.^11, 12^ It is also a key factor in the resistance of malignant cells to ABT-737 and navitoclax. Moreover, MCL-1 silencing has repeatedly been found to elicit tumor regression and cell death in various models. Therefore, a highly specific MCL-1 inhibitor would be greatly valuable in malignancies for which survival depends mainly or partly on MCL-1. The approach of inhibiting MCL-1 expression has been developed with cyclin-dependent kinase inhibitors (e.g., flavopiridol, roscovotine and dinaciclib) but their action is indirect and non-specific.^13^ Several small molecules and BH3 peptides capable of directly binding MCL-1 and antagonizing its activity have been characterized such as MIM-1 and small molecule Mcl-1 inhibitor.^14^ However, none of them are considered as *bona fide* BH3 mimetics. Actually, high binding affinity is difficult to achieve, which may be due to the nature of the protein/protein interaction and the conformational rigidity of the hydrophobic binding groove of MCL-1.^15^ There are also difficulties to assess the mechanism of action. This gap just comes to be filled with the discovery of A-1210477 and related analogs.

Bruncko *et al.*^16^ first described a series of MCL-1 inhibitors derived from an indole-2-carboxylic acid core by high-throughput screening and structure-guided design. The compounds bind to MCL-1 with sub-nanomolar affinity (0.45 nM) and excellent selectivity over other BCL-2 family proteins. They have a 100-fold higher affinity than the indole-2-carboxylic acid previously identified by Friberg *et al.*^17^ (using NMR-based fragment screen approach): indeed, this small molecule Mcl-1 inhibitor has a binding affinity of 55 nM (which seems too low to confer on-target cellular effects). In a thorough mechanistic study on the series of MCL-1 inhibitors from Bruncko *et al.*,^16^ Leverson *et al.*^1^ have shown that the compound A-1210477 and several related analogs disrupt MCL-1-BIM and MCL-1-NOXA complexes. In contrast, no effect was observed with another analog, which binds MCL-1 with a low affinity (130 nM) comparable to that of MCL-1 inhibitors described elsewhere. By using different methods and experimental conditions, Leverson *et al.*^1^ also demonstrated that A-1210477 and its related analogs penetrate living cells and act directly on their target MCL-1. The compounds trigger apoptosis in multiple myeloma and non-small cell lung cancer cell lines that have been validated to be MCL-1-dependent. They induce hallmarks of the intrinsic pathway of apoptosis (mitochondrial membrane depolarization, cytochrome *c* release, caspase-3/7 activation and caspase-dependent phosphatidylserine externalization at the cell surface). In addition, apoptosis induction depends on BAX/BAK activation, confirming that the compounds act through an on-target mechanism. Consequently, A-1210477 and related analogs are the first *bona fide* BH3 mimetics specific for MCL-1 (Table 1). Furthermore, A-1210477 synergizes with navitoclax to induce apoptosis in various cancer cell lines, indicating that the MCL-1-selective inhibitors might circumvent the resistance to navitoclax (or ABT-199) when used in combination.^1^

An important issue is whether normal tissues could tolerate MCL-1 inhibition at the level required for therapeutic benefit. Indeed, MCL-1 knockout models suggest a number of toxic side effects. However, it is possible that inhibiting the functional activity of MCL-1 could be less toxic than the complete loss of the protein.

Whereas derivatives with appropriate pharmacologic properties are needed for the clinical application, the discovery of A-1210477 and related small molecules already appears as a great step forward in overcoming the therapeutic resistance of cancers that are at least in part dependent on MCL-1. Thus, these latest data on BH3 mimetic research are undoubtedly crucial to advance the targeted treatment of patients.

## Figures and Tables

**Table 1:** Comparison of A-1210477 and related analogs with previous small-molecule inhibitors of prosurvival BCL-2 family proteins acting as authentic BH3 mimetics

**Compounds**	**Specific targets**	**Status**
ABT-737^5^	BCL-2, BCL-XL and BCL-W	Preclinical
Navitoclax^6^	BCL-2, BCL-XL and BCL-W	Phase II trials^7^
ABT-199^8^	BCL-2	Phase III trials^7^
BM-1197^9^	BCL-2 and BCL-XL	Preclinical
A-1155463^10^	BCL-XL	Preclinical
A-1210477 and analogs^1, 16^	MCL-1	Preclinical
